# Microbial fuel cell assisted band gap narrowed TiO_2_ for visible light-induced photocatalytic activities and power generation

**DOI:** 10.1038/s41598-018-19617-2

**Published:** 2018-01-29

**Authors:** Mohammad Ehtisham Khan, Mohammad Mansoob Khan, Bong-Ki Min, Moo Hwan Cho

**Affiliations:** 1 School of Chemical Engineering, Yeungnam University, Gyeongsan-si, Gyeongbuk 38541 South Korea; 2 Chemical Sciences, Faculty of Science, Universiti Brunei Darussalam, Jalan Tungku Link, Gadong, BE 1410 Brunei Darussalam; 3 Materials Science Centre, Yeungnam University, Gyeongsan-si, Gyeongbuk 38541 South Korea

## Abstract

This paper reports a simple, biogenic and green approach to obtain narrow band gap and visible light-active TiO_2_ nanoparticles. Commercial white TiO_2_ (*w*-TiO_2_) was treated in the cathode chamber of a Microbial Fuel Cell (MFC), which produced modified light gray TiO_2_ (*g*-TiO_2_) nanoparticles. The DRS, PL, XRD, EPR, HR-TEM, and XPS were performed to understand the band gap decline of *g*-TiO_2_. The optical study revealed a significant decrease in the band gap of the *g*-TiO_2_ (E_*g*_ = 2.80 eV) compared to the *w*-TiO_2_ (E_*g*_ = 3.10 eV). The XPS revealed variations in the surface states, composition, Ti^4+^ to Ti^3+^ ratio, and oxygen vacancies in the *g*-TiO_2_. The Ti^3+^ and oxygen vacancy-induced enhanced visible light photocatalytic activity of *g*-TiO_2_ was confirmed by degrading different model dyes. The enhanced photoelectrochemical response under visible light irradiation further supported the improved performance of the *g*-TiO_2_ owing to a decrease in the electron transfer resistance and an increase in charge transfer rate. During the TiO_2_ treatment process, electricity generation in MFC was also observed, which was ~0.3979 V corresponding to a power density of 70.39 mW/m^2^. This study confirms narrow band gap TiO_2_ can be easily obtained and used effectively as photocatalysts and photoelectrode material.

## Introduction

Since 1972, titanium dioxide (TiO_2_) has been recognized as a potential photocatalyst by researcher’s worldwide^1^.TiO_2_ nanocrystals are well-known semiconductors that can catalyze solar powered reactions, such as water splitting, catalysis, photocatalysis, and environmental remediation^2–5^. On the other hand, TiO_2_ mainly absorbs light in the UV part of the solar spectrum; therefore, researchers hope that lowering its band gap energy will also enable it to absorb visible and infrared light^4–9^.

Previously studies have reduced the band gap of TiO_2_ by doping it with metals or non-metals, wrapping it with graphene, or introducing intrinsic defects into the TiO_2_ crystals^10–22^. These methods somehow increase the amount of partial visible light absorption but complete visible light and infrared light absorption is not achieved. Researchers used hydrogenation and other techniques to produce a black, red, blue form of TiO_2_ by introducing disorder, which absorbs light in the UV, visible and near infrared regions of the spectrum^6–9^. This increases the amount of solar light absorption of the black, red, and blue TiO_2_, which can be used to generate hydrogen gas and be applied in other visible light-induced applications, such as environmental remediation^6–9,23,24^. Researchers have also found that the hydrogenation process produces disorder in the surface layer of the TiO_2_ nanocrystals. Based on these studies, researchers have suggested that the hydrogen also ‘mops up’ broken titanium and oxygen bonds, forming new bonds that lower the band gap to the near infrared region^23–28^.

Significant developments in TiO_2_ photocatalysis and hydrogenation as well as other approaches that are novel and unique, such as surface doping or modification of TiO_2_, have been made to increase the photocatalytic activity under visible light irradiation^25,26^. Black TiO_2_ was reported to catalyze the photo-decomposition of organic molecules much better than normal nanophase TiO_2_^26^. They also found that its ability to catalyze water splitting into hydrogen and oxygen under sunlight was improved greatly. Compared to conventional TiO_2_ and other metal oxide materials, it exhibits significantly higher efficiency under the same conditions^6^.

We previously reported band gap engineered TiO_2_ nanoparticles using electron beam irradiation which was simple and the reproducibility was quite high. Electron beam irradiated TiO_2_ nanoparticles showed notable decrease in the band gap as well as enhanced visible light-induced photo-degradation response towards methylene blue (MB) and brilliant blue G (BB) degradation, which was not possible for untreated TiO_2_ nanoparticles under similar conditions^21^. In the present study, white commercial TiO_2_ (*w*-TiO_2_) was modified (by forming defects) to a light gray colour (*g*-TiO_2_) using the cathode chamber of a Microbial Fuel Cell (MFC), which is a green, biogenic, novel and energy efficient process as compared to electron beam irradiation approach. The *g*-TiO_2_ showed an absorbance in the visible and near infrared region of the solar spectrum as well as improved visible light-induced photocatalytic and photoelectrochemical performances. The enhanced visible light activities of *g*-TiO_2_ might lead to various novel and efficient applications, which will open new horizons for metal oxide nanostructures with different types of defects and narrow band gap energies. Surprisingly, the treatment of TiO_2_ in the MFC cathode also generated electricity, which is energy efficient. The main difference between electron beam irradiation approach^21^ and MFC treatment is that, this approach is environmentally friendly approach which does not involve any external energy, chemicals or doping agents which make this modification method highly economical, simple, green, biogenic, useful, and efficient in the field of band gap engineering of metal oxides and has great potential for real applications in the photodegradation of several toxic dyes.

## Results and Discussion

This paper reports a novel and alternative methodology to improve the visible light absorption of *w*-TiO_2_ by engineering it and forming different types of disorder. The modification process was achieved in MFC, which can produce an array of defects or disorder in the TiO_2_ nanoparticles, by this means, imparting novel characteristics, such as a reduced band gap, rapid charge carrier movements, and visible light-induced photocatalytic activities. This easy synthesis procedure does not include any expensive, toxic and hazardous chemicals, which make this modification method highly economical, simple, green, useful, and efficient in the field of band gap engineering of metal oxides. The complete modification process takes place in water at room temperature under atmospheric pressure. Fig. S1 presents the schematic diagram aimed at the modification of commercially obtainable TiO_2_ nanoparticles in a MFC. The electrons and protons generated in the anode of the MFC can either interact with some of the Ti^4+^ ions and reduce them to Ti^3+^ or interact with the TiO_2_ surface, which can alter the TiO_2_ composition^21,29^. The *in-situ* created species, such as electrons, protons, and Ti^3+^ ions, modified the TiO_2_ surface, which may impart enhanced visible light induced photocatalytic activities to *g*-TiO_2_^21,30,31^.

### X-ray diffraction studies

**Figure 1:**
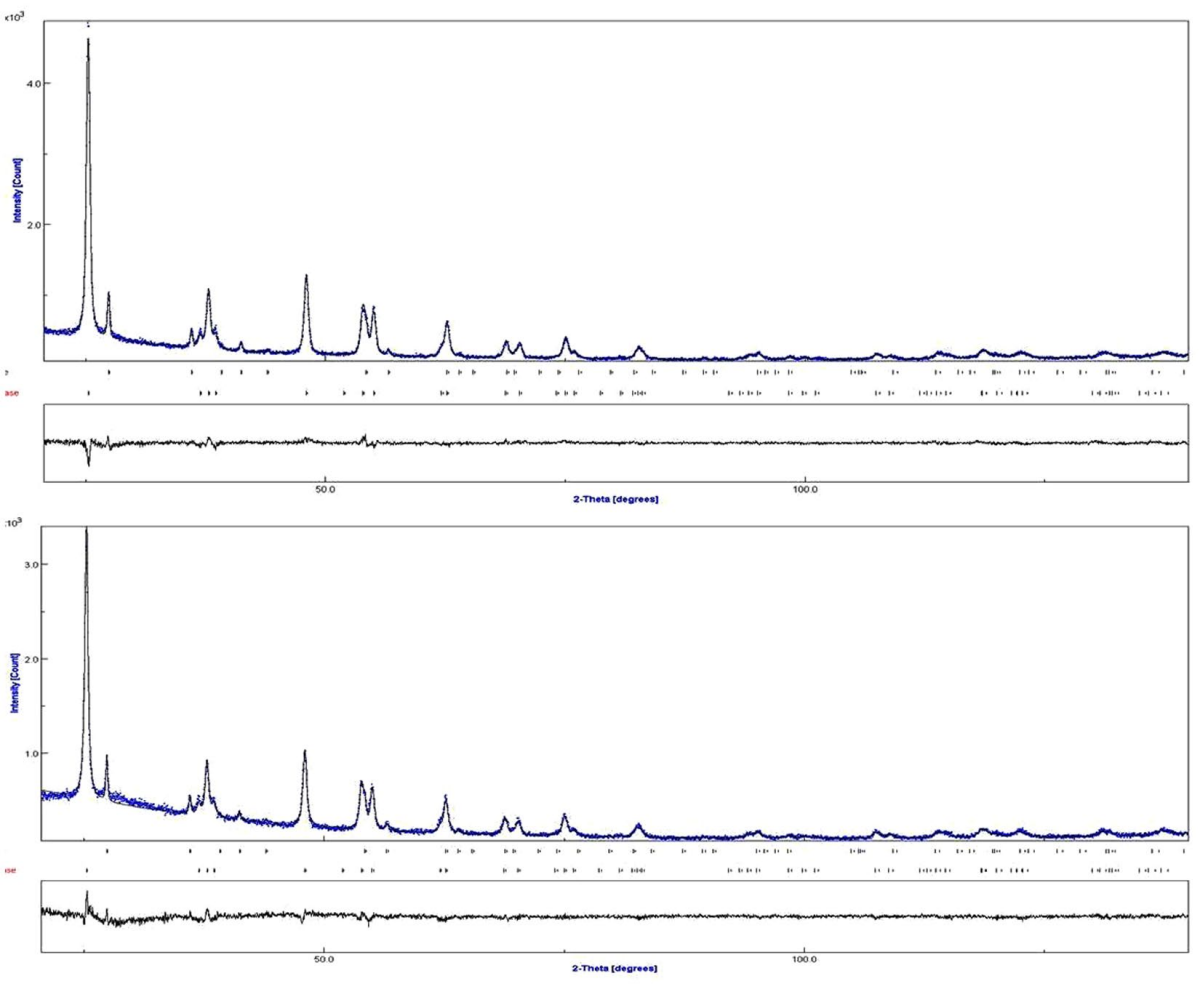
XRD patterns of the (**a**) *w*-TiO_2_, and (**b**) *g*-TiO_2_ nanoparticles obtained after Rietveld refinement of *w*-TiO_2_ and *g*-TiO_2_.

The structures of the *w*-TiO_2_ and *g*-TiO_2_ nanoparticles were examined by XRD in the 10–140° 2θ range. The strong XRD peaks (Fig. 1) indicate that the TiO_2_ nanoparticles were highly crystalline. The crystalline phase had an anatase and a rutile structure (Table 1) with a mean crystal size of approximately 25 nm, which is in agreement with the HRTEM observations.

**Table 1:** Relevant crystallographic data for *w*-TiO_2_ and *g*-TiO_2_ nanoparticles established from the powder X-ray diffraction data after Rietveld refinements.

Sample	Phase	Phase fraction	Cell parameter(Å)			V(Å^3^)
			a	b	c	
*w*-TiO_2_	Anatase	0.908	3.7890	3.7890	9.5131	136.58
	Rutile	0.092	4.5989	4.5989	2.9583	62.586
*g*-TiO_2_	Anatase	0.901	3.7896	3.7896	9.5127	136.61
	Rutile	0.099	4.5985	4.5985	2.9620	62.635

The Rietveld refinement was used as a simple tool to verify the precise structure of the *w*-TiO_2_ and *g*-TiO_2_ nanoparticles and analyse the phase transformation from anatase to rutile. Using this method, the structural compositions of each samples were analysed qualitatively by fitting the experimental powder XRD profiles with respect to corresponding structural parameters (i.e. lattice parameters, atomic coordinates) and instrumental parameters (i.e. zero-point and profile parameters), as shown in Table 1. The refinement was converted to final residual factors (Table S2) of R_wp_ = 7.59 ± 0.3%, R_exp_ = 6.83 ± 0.2% and χ^2^ = 1.234 ± 0.2 for *w*-TiO_2_ nanoparticles, and R_wp_ = 7.70 ± 0.2%, R_exp_ = 6.69 ± 0.1% and χ^2^ = 1.324 ± 0.3 for *g*-TiO_2_ nanoparticles. R_wp_ is the weighted-profile R value, R_exp_ is the statistically expected R value, and χ^2^ is the goodness of fit (GoF), which is the square of the ratio between R_wp_ and R_exp_. The χ^2^ is the goodness of fit values was calculated using given below equation (1). These calculated values of Rietveld refinement further confirmed the changes/modification of the *w*-TiO_2_ to *g*-TiO_2_ nanoparticles^32^.

1 $$GoF=\frac{\sum _{i}{w}_{i}{({y}_{io}-{y}_{ic})}^{2}}{N-P}={(\frac{{R}_{wp}}{{R}_{\exp }})}^{2}$$

where, N is the number of points and P is the number of parameters.

**Figure 2:**
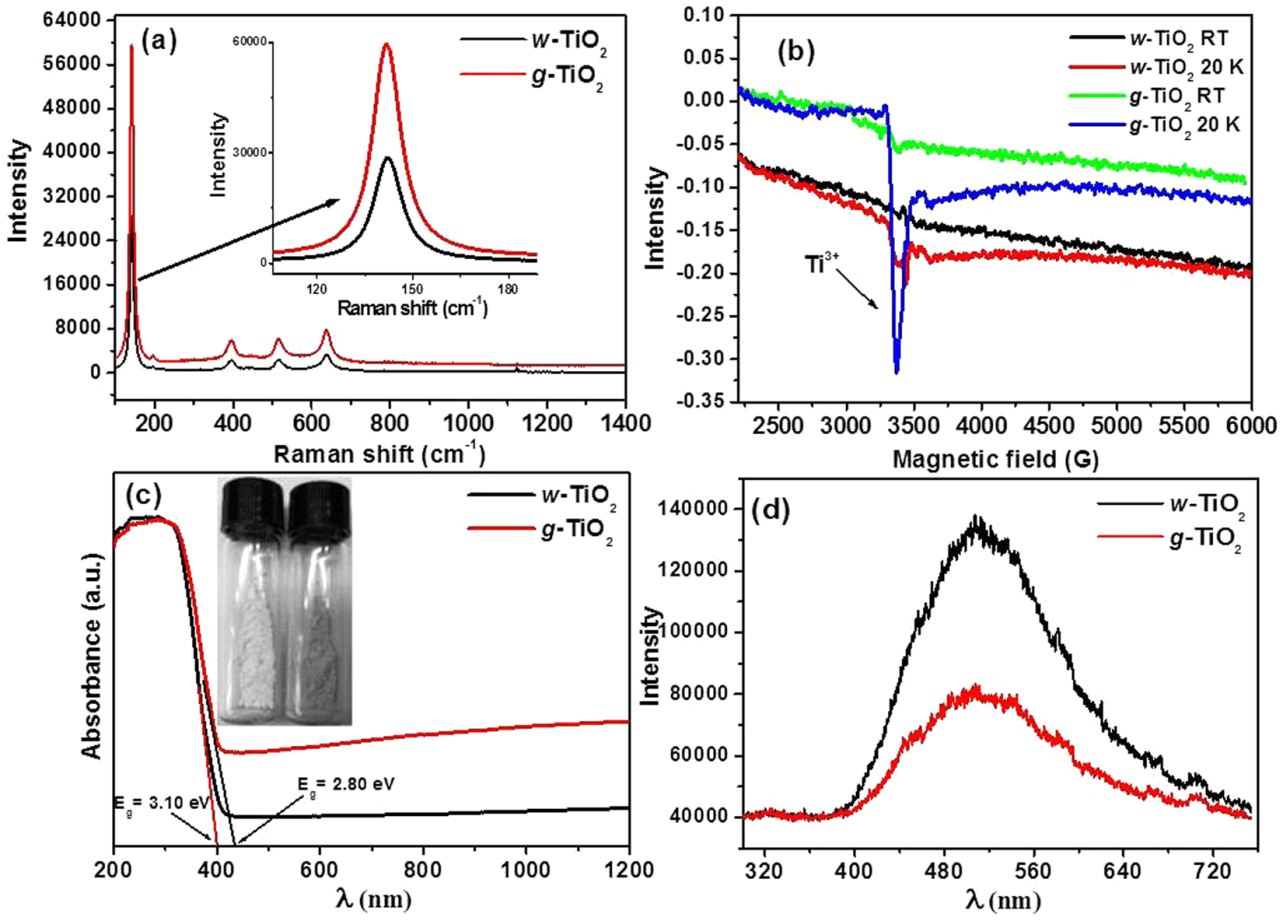
(**a**) Raman spectra, (**b**) EPR spectra at room temperature and 20 K, (**c**) UV-vis diffuse absorbance spectra, and (**d**) PL spectra of the *w*-TiO_2_ and *g*-TiO_2_ nanoparticles.

The Rietveld refinement of the XRD data of the *w*-TiO_2_ and *g*-TiO_2_ nanoparticles showed that after modification, the phase fraction of anatase of *w*-TiO_2_ decreased 1.234 ± 0.2 compared to *g*-TiO_2_ nanoparticles, whereas the phase fraction of rutile of *w*-TiO_2_ was increased to 1.324 ± 0.3 compared to *g*-TiO_2_ nanoparticles. The phase fraction decrease (anatase) and increase (rutile) of *g*-TiO_2_ compared to *w*-TiO_2_ was attributed to the oxidation state changes from Ti^4+^ to Ti^3+^ which was confirmed from these values (1.234 ± 0.2) and 1.324 ± 0.3). It addition, the phase fraction is also indicated by the unit cell volume of *g*-TiO_2_ nanoparticles for the anatase and rutile phase increased ~0.02% and ~0.106% compared to *w*-TiO_2_ (Table 1). The increase in the unit cell volume was attributed to the formation of some Ti^3+^ and defects in the sample, which is expected to be responsible for the increase in unit cell volume^21,33^. This XRD technique is incompatible to distinguish the oxidation state changes of two samples. The formation of Ti^3+^ can be easily detected by using EPR analysis (Fig. 2(b)) which is the confirmation of Ti^3+^formation in gray titania (Fig. 1).

### Raman spectroscopy

Raman spectroscopy was performed to observe the variations in the structure and disorder in the *g*-TiO_2_ nanoparticles after the introduction of disorder using MFC. The two polymorphs of TiO_2_ belong to different space groups: D_4h_^19^ (*I4*_1_/*amd*) for anatase and D_4h_^14^ (*P4*_2_*/mnm*) for rutile, which has distinct characteristics in Raman spectra^6^. For anatase TiO_2_, there are six Raman active modes with frequencies at 144, 197, 399, 515, 519 (superimposed with the 515 cm^–1^ band), and 639 cm^–1^ ^6^. The modified *g*-TiO_2_ nanoparticles display the typical anatase Raman bands similar to the *w*-TiO_2_ nanoparticles, in addition to the increased intensity peak of the anatase Raman peak (Fig. 2a). The characteristic anatase mode at 144 cm^–1^ was present in both samples (*w*-TiO_2_ and *g*-TiO_2_) which indicate dominance of the anatase type TiO_2_ presence in the samples. Das *et al.*^33^ and Park *et al.*^34^ reported that the intensity of the peak increases with increasing number of non-stoichiometry defects, which increase the light absorption capacity of the TiO_2_ nanoparticles. Therefore, in the present case the increased intensity of the Raman bands clearly indicates that structural changes have occurred after a treatment in MFC, resulting in formation of some disorder or defects, which were confirmed further by EPR and DRS analysis^21,33–36^.

### Electron paramagnetic resonance

The EPR spectra of the *w*-TiO_2_ and *g*-TiO_2_ nanoparticles were recorded (Fig. 2b) at room temperature (RT) and 20 K. The *w*-TiO_2_ and *g*-TiO_2_ nanoparticles at RT did not show any EPR signals, while the EPR signals for *w*-TiO_2_ and *g*-TiO_2_ nanoparticles were clear at 20 K. The EPR signal for *g*-TiO_2_ at 20 K was much stronger than that of *w*-TiO_2_ with a *g* value of 2.04^28^. The detected *g* value (Fig. S2) matched the characteristics of the paramagnetic Ti^3+^ ion centre in a distorted rhombic oxygen ligand field. The *w*-TiO_2_ showed another EPR signal at 20 K, which corresponds to oxygen vacancies, which was very small, whereas in the case of *g*-TiO_2_, this EPR signal appears to overlap with the Ti^3+^ signal, resulting in dramatic enhancement of the peak intensity^8,29,30^. Therefore, EPR showed that *g*-TiO_2_ has paramagnetic characteristics and oxygen vacancies, which enhances the visible light-induced photocatalytic activity^8,31,37,38^. Note that EPR is insensitive to Ti^4+^ species; hence, no signal is expected from it. This observation is very useful for identifying the Ti^3+^-related defects and oxygen vacancies related with Ti^3+^ lattice and F^+^ centres^20^ (Fig. 2).

### UV-vis spectroscopic studies

Figure 2c shows the UV-vis diffuse absorbance spectra, i.e., optical response of the *w*-TiO_2_ and *g*-TiO_2_ nanoparticles, in which *g*-TiO_2_ nanoparticles show higher visible-light absorption than the *w*-TiO_2_ nanoparticles. The commercial *w*-TiO_2_nanoparticles only respond to ultraviolet light due to its intrinsic wide band gap. The improvement in the visible-light absorption of *g*-TiO_2_ nanoparticles was attributed to two factors: the formation of oxygen vacancies^8,17^, and surface disorder^8,16^. Mao *et al*. reported the surface disorder of anatase TiO_2_ nanoparticles following a hydrogen treatment, which shifted the valence band position by 2.18 eV^16^. In this case, the band gap of *w*-TiO_2_ nanoparticles shifted from 3.15 eV to 2.80 eV (Fig. 2c) for *g*-TiO_2_ nanoparticles. As a result, the energy gap between the valence band and the conduction band was narrowed dramatically to the point that was small enough for visible-light absorption. Hence, it was attributed to the absorption of visible light because of the formation of oxygen vacancies and other related defects in *g*-TiO_2_. The energy levels of the oxygen vacancies are approximately 0.75–1.18 eV below the conduction band of hydrogen-reduced TiO_2_^8,20^. The visible-light absorption is associated with the transitions from the TiO_2_ valence band to the oxygen vacancy levels or from the oxygen vacancies to the TiO_2_ conduction band^20–22^. For the *g*-TiO_2_ nanoparticles, which appears to be self-doped Ti^3+^ (Fig. 2(c), red line), the considerably large absorption tail in the visible and NIR regions was observed, which is consistent with the change in colour of the powders from white to gray (Fig. 2c)^8,9,21^. The high absorption tail in the visible and NIR regions also provides clear evidence that the *g*-TiO_2_ nanoparticles contain a large number of oxygen vacancies^8,9^. The high absorption tail in the visible and NIR regions also provides clear evidence that the *g*-TiO_2_ nanoparticles contain a large number of defects/oxygen vacancies^6,24^.

These results also suggest that Ti^3+^ induced visible-light absorption that would have formed an isolated states between the forbidden gap in TiO_2_, as reported previously, rather than a shift in the position of either band edge, which usually takes place as a result of doping with metals or non-metals^2,12–16,39^. Theoretical studies also confirmed that formation of oxygen vacancies and Ti^3+^ could result in an electronic state vacancy band below the conduction band^8,21^.

### PL studies

PL was performed to understand the migration, and transfer of charge carriers, efficiency of charge carrier trapping in semiconductor nanostructures. It is well identified that the PL signals of semiconductor materials result from the recombination rate of photo-induced charge carriers. In general, the lower the PL intensity, the lower the recombination rate of photo-induced electron–hole pairs, and the higher the photocatalytic activity of semiconductor photocatalysts^39^. In photoluminescence signals and their intensity are closely related to the improved photocatalytic activity. Further addition or modification in metal NPs reduces the PL intensities, due to shorter distance of inter band metal ions, which result in an energy transfer between nearby ions. The technique is useful because PL emission occurs mainly through the recombination of free carriers. The PL spectra of semiconductors are related to the transfer behaviour of the photo-induced electrons and holes, and can be used to estimate the recombination rate of charge carriers and understand the fate of electron–hole pairs in semiconductor nanostructures^40,41^. In other words, the PL emission intensity is generally associated with the recombination rate of the photo-induced electrons and holes, in which higher emission intensity reflects the fast recombination rate, whereas a lower intensity reflects the relatively slow recombination rates. This lower recombination may provide large number of charge carriers, which is actively participating in a range of oxidative and reductive photocatalytic degradation reactions of various dyes. As shown in Fig. 2d, the PL emission intensity of *w*-TiO_2_ was reduced significantly after inducing defects by MFC, which suggests that the charge carriers are trapped by the defects present in the *g*-TiO_2_, which enhances the charge separation efficiency. Different types of defects were reported to greatly affect the PL emission intensity of the metal oxides. For example, Jing *et al*.^39^, and Chetri *et al*.^41^, reported that the presence of defects quenched the PL signals of metal oxides significantly. Based on the above discussion, it could be concluded that the presence of defects in the *g*-TiO_2_ acts as trapping centres, which reduces the emission intensity of the PL signal^40^.

### Microstructure analysis of w-TiO2 and g-TiO2 nanoparticles

**Figure 3:**
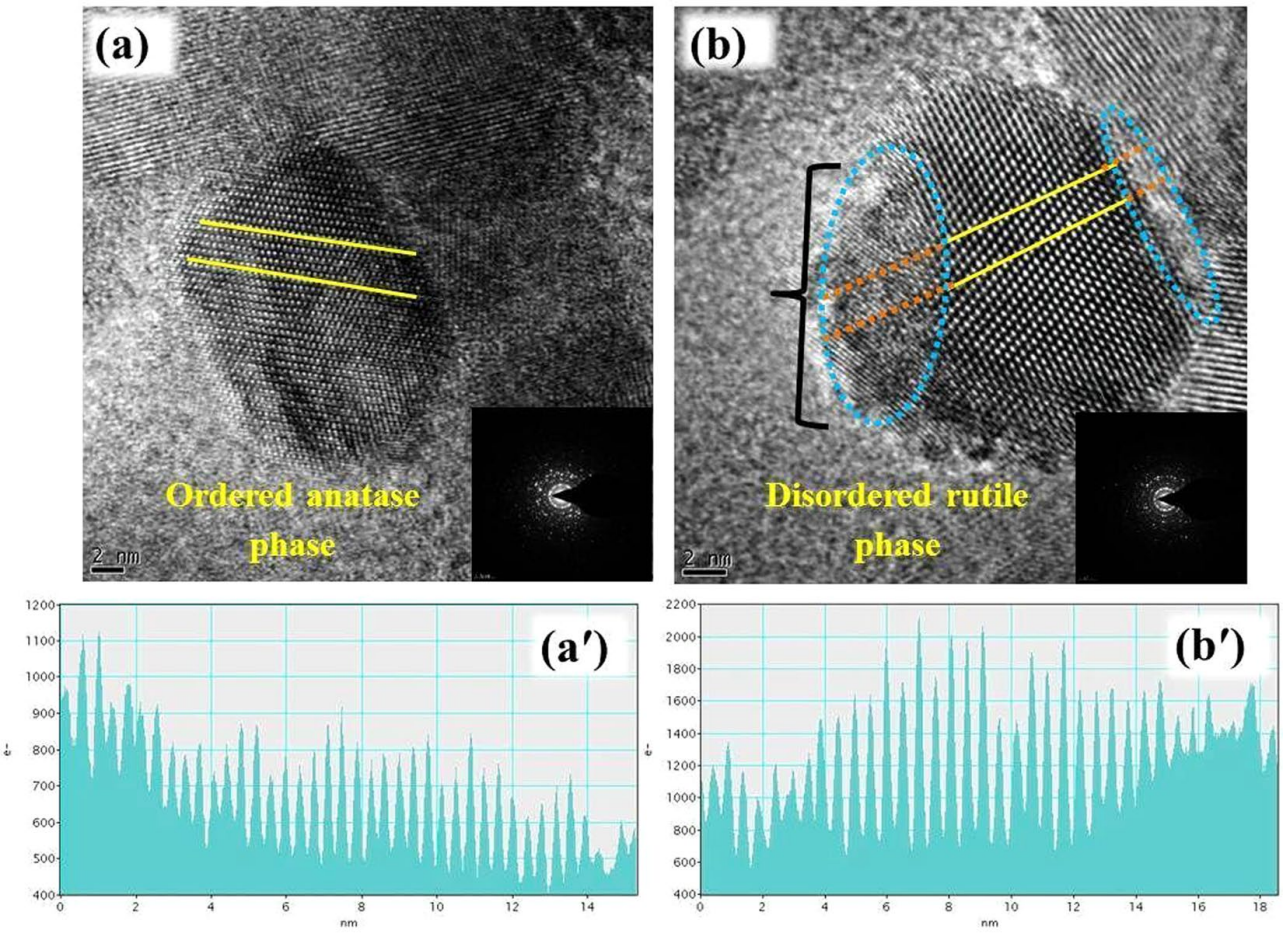
HRTEM image and structural analysis of (**a**) *w*-TiO_2_ and (**b**) *g*-TiO_2_. The insets in **(a)** and **(b)** show the corresponding selected area electron diffraction pattern. (**a′**) shows line analysis of *w*-TiO_2_ and (**b′**) shows line analysis of *g*-TiO_2_. The zeros of the axis in (a′) and (b′) correspond to the left ends of the lines in (**a**) and (**b**).

Figure 3 shows HRTEM images, SAED, and structural analysis of the *w*-TiO_2_ and *g*-TiO_2_ nanoparticles, which are in the range of 15 to 30 nm and in accordance with XRD analysis. *w*-TiO_2_ was completely crystalline, showing clearly-resolved and well-defined lattice fringes, even at the surface of the nanocrystals (Fig. 3a). The distance between the adjacent lattice planes was 0.37 nm, which is typical for anatase, and uniform throughout the whole nanocrystals (Fig. 3a). The measured lattice spacing of 0.37 nm coordinated the distance between the {101} planes of the anatase TiO_2_ crystal. The reflection from the similar {101} plane was prominent in the XRD patterns (Fig. 1) of the *w*-TiO_2_ and *g*-TiO_2_ nanoparticles. The spot SAED pattern [inset of Fig. 3(a),(b)] and continuous lattice confirmed the crystalline nature of the *w*-TiO_2_ and *g*-TiO_2_ nanoparticles. On the other hand, the outer edge of *g*-TiO_2_ nanoparticles gives the blurry impression, indicating an amorphous or disorder phase on the nanoparticles surface. The *g*-TiO_2_nanoparticles has a crystalline-disordered structure (Fig. 3b), and the outer layer can be seen readily, which is a structural deviation from the standard crystalline anatase. In contrast, the yellow colour encircled lattice line is disrupted and unclear at the edge of the nanoparticles. The core of the nanoparticles shows a well resolved {101} lattice plane with typical anatase plane distance on the disordered outer layer; the distances between the adjacent lattices planes are no longer uniform (Fig. 3b). This structural difference clearly verifies the disordered crystalline nature of the surface layer of the *g*-TiO_2_. Moreover, HRTEM of the *w*-TiO_2_ and *g*-TiO_2_ nanoparticles did not show any distinct changes in crystallinity (Fig. 3).

**Figure 4:**
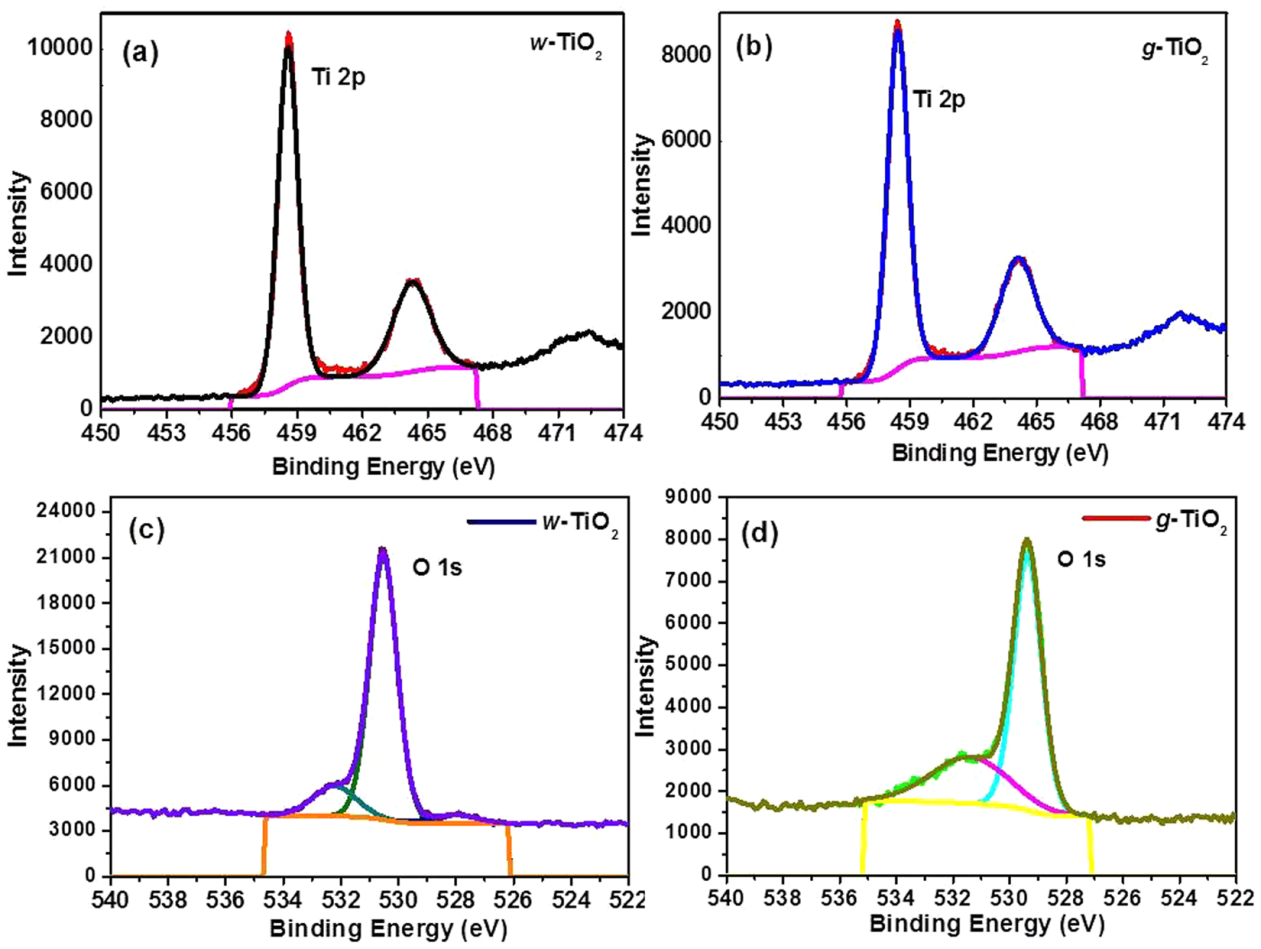
XPS (**a** and **b**) Ti 2p, and (**c** and **d**) O 1 s of the *w*-TiO_2_ and *g*-TiO_2_ nanoparticles.

Figure. 3(a′) shows the distance between the adjacent lattice planes, in the nm range, which is characteristic of anatase and uniform throughout the entire nanocrystals spectrum of *w*-TiO_2_ and (b′) displays a crystalline-disordered core-shell structure for *g*-TiO_2_ nanoparticles. Fig. S3(a), (b) as well as Fig. S3(a′), (b′) shows the HAADF-STEM images and EDX data of the *w*-TiO_2_ and *g*-TiO_2_ nanoparticles. In contrast, the difference between Fig. S3a,b suggests the surface modification of *w*-TiO_2_ in MFC. This finding was confirmed by SAED (the inset of Fig. 4a,b) and EDX analysis (Fig. S3a′,b′) of different regions of the nanoparticles.

### XPS Analysis

XPS was performed on *w*-TiO_2_ and *g*-TiO_2_ nanoparticles for the surface characterization, oxidation states and other observations. C, O, and Ti were noticed in the survey scan spectra (Fig. S4(a) ESI). The C 1 s photoelectron peak (Fig. S4(b) ESI) at a binding energy (BE) of 284.5 eV was stronger for *w*-TiO_2_ than the *g*-TiO_2_ nanoparticles, which was ascribed to the elimination of surface carbon impurities from the *g*-TiO_2_ nanoparticles. Figure 4(a,b) shows the XP spectra of *w*-TiO_2_ and *g*-TiO_2_, respectively, in the Ti 2p BE region. The XPS Ti 2p peak was deconvoluted into two Ti 2p peaks at 458.57 and 464.27 eV for *w*-TiO_2_, whereas the peaks were observed at 458.42 and 464.10 eV for *g*-TiO_2_ nanoparticles, which were attributed to the splitting of Ti into Ti 2p^3/2^ and Ti 2p^1/2^ ^7,41,42^. Both Ti 2p^3/2^ and Ti 2p^1/2^ peaks shifted towards a lower binding energy in the case of *g*-TiO_2_, which confirms the modification and formation of Ti^3+^ in the TiO_2_nanostructurein MFC setup. Similarly, Zhao *et al*.^43^, also reported that lower shift of Ti 2p BE is due to the formation of Ti^3+^ in TiO_2_ lattices. The amount of Ti^3+^ on the TiO_2_ surface plays a significant role, as described in the case of TiO_2_ doped with metal atoms. The photogenerated electrons can be confined in Ti^3+^, thus preventing the recombination rate of majority and minority carriers^43^. To control the binding states of oxygen in *w*-TiO_2_ and *g*-TiO_2_, the O 1 s XPS peak was fitted to three peaks (Fig. 4(c),(d)) cantered at 530.52, 532.22, and 528.06 eV for *w*-TiO_2_ and 530.62, 531.92, and 529.68 eV for *g*-TiO_2_^6,42–44^. The shift in the O 1 s BE of *g*-TiO_2_ compared to *w*-TiO_2 _specifies a modification in the form of oxygen bonding, which is associated to the creation of Ti^3+^ ^8^. The photoelectron peak sat around 530.62 and 529.68 eV were allocated to the lattice oxygen in TiO_2_ and Ti_2_O_3_, correspondingly, however the peak at 531.92 eV was allotted to the water adsorbed on the TiO_2_ surface (Fig. 4).

**Figure 5:**
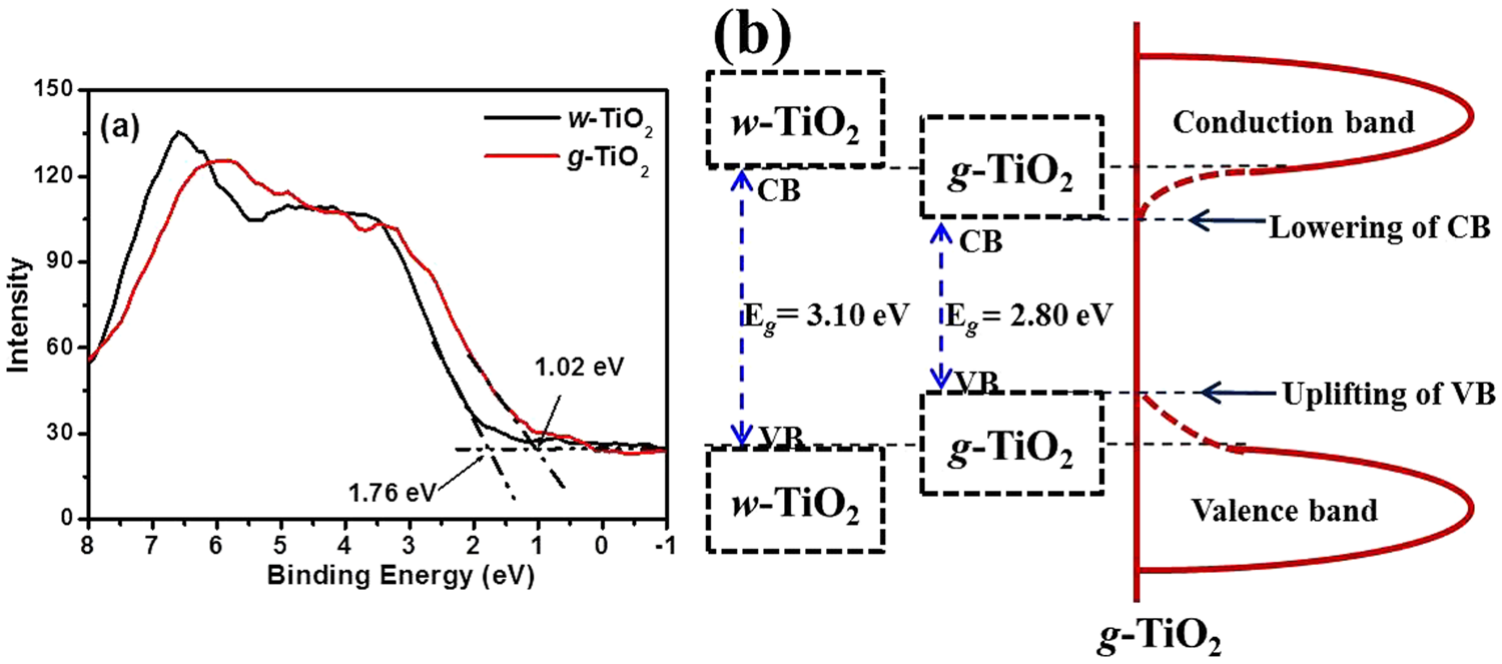
(**a**) VB of the *w*-TiO_2_ and *g*-TiO_2_ nanoparticles, and, (**b**) Proposed DOS for the *g*-TiO_2_ nanoparticles.

The reduction in the band gap may take place through the development of mid-gap band states whichever below the conduction band (CB) or above the valence band (VB) overlying with the respective bands. Therefore, VB XPS of the *w*-TiO_2_ and *g*-TiO_2_ nanoparticles was achieved to observe the band gap reduction phenomenon (Fig. 5(a)). The VB maximum of *w*-TiO_2_ was detected at 1.76 eV, whereas the VB maximum of the *g*-TiO_2_ was observed at 1.02 eV, showing a 0.74 eV shifts to a lower binding energy^6,18,45^. This shift was assigned to surface oxygen vacancies, Ti^3+^ formation and/or disorderliness in accordance with the TEM result and several other recent reports^6,18,21,28^. In particular, the band gap reduction caused by a lowering of CB was reported due to defects, such as oxygen vacancies and Ti^3+^ formation, which is related mainly to oxygen vacancies^2,16,18,28,45^. Chen *et al*. reported such an increase in the VB, which was due mainly to the existence of a disorder shell in the hydrogenated black TiO_2_ nanoparticles^6^. The reduction of the band gap in the *g*-TiO_2_ case was attributed to both the dropping of CB (due to oxygen vacancies and Ti^3+^ defect centres) and the increase in the VB (due to surface disorderliness)^2,6,16,18,28^.

Based on the VB XPS results (Fig. 5(a),(b) presents a schematic illustration of the density of states (DOS) of disorder-engineered *g*-TiO_2_ nanoparticles compared to those of unmodified *w*-TiO_2_ nanoparticles. A measured band gap of 3.10 eV indicates a negligible change in the band edges of *w*-TiO_2_. The *w*-TiO_2_ displayed the typical VB DOS characteristics of TiO_2_, with the edge of the maximum energy at approximately 1.76 eV. Therefore, the CB minimum would occur at ~1.50 eV. For *g*-TiO_2_, the VB maximum energy showed a blue-shift toward the vacuum level at ~1.02 eV. A lower band gap from the DRS measurement for the *g*-TiO_2_ and VB XPS shift was due to the surface disorder produced after the MFC treatment. In addition, there may be CB tail states arising from the defects (Ti^3+^) that extend below the conduction band minimum^2,6,16,18,28^. The optical transitions from the blue-shifted VB edge to the band tail states are apparently responsible for optical absorption in *g*-TiO_2_. This assumption was supported by DRS observations. An additional potential advantage of this engineered and disordered *g*-TiO_2_ is that such defected and disordered metal oxides provide trapping sites (such as Ti^3+^) for photogenerated carriers and inhibit them from rapid recombination, thereby enhancing electron transfer and photocatalytic reactions^21,29,30^ (Fig. 5).

### Photoelectrochemical studies

**Figure 6:**
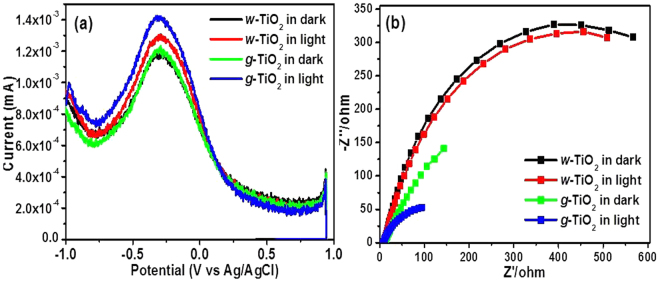
(**a**) DPV and (**b**) EIS of the *w*-TiO_2_ and *g*-TiO_2_ nanoparticles.

DPV is generally used to determine the charge storage capability of nanomaterials and is used frequently as a complementary technique to cyclic voltammetry. DPV was performed to understand the charge storage capability and the quantized behaviours of the *w*-TiO_2_ and *g*-TiO_2_ nanoparticles^46–49^. Figure 6a shows the well-defined quantized capacitance charging peaks for the *w*-TiO_2_ and *g*-TiO_2_ nanoparticles in the dark and under visible light irradiation. The peak current for *w*-TiO_2_ and *g*-TiO_2_ nanoparticles in the dark was observed at 1.170 mA and 1.218 mA, whereas, it was observed at 1.290 mA and 1.416 mA under visible light, respectively. An increase in the peak current of 0.126 mA was observed in the case of *g*-TiO_2_ nanoparticles under visible light irradiation. For the peak potential of *w*-TiO_2_ and *g*-TiO_2_ nanoparticles, no shift was observed. The increase in the peak current (0.126 mA) clearly shows the enhancement in the photoelectrochemical activity of *g*-TiO_2_ nanoparticles compared to the *w*-TiO_2_ nanoparticles, which may be due to the different types of defects formed in *g*-TiO_2_ nanoparticles. The *g*-TiO_2_ nanoparticles under visible light irradiation exhibited excellent and enhanced charge storing properties compared to *w*-TiO_2_ nanoparticles. The electrons stored on the *g*-TiO_2_ nanoparticles could be used to form different oxidative species (O_2_^•^ and ^•^OH) under visible light irradiation. These highly oxidative species might be responsible for degradation and mineralization of the organic colored dyes^21,29,30^. In addition, these stored electrons can be used for different photoactive devices. Overall, the *g*-TiO_2_ nanoparticles could be a good photoelectrocatalyst for electron transfer reactions such as photocatalysis and optoelectronic devices (Fig. 6).

Generally, EIS is used to examine the electrochemical properties of the materials; hence, it was performed to understand the charge transfer resistance and charge separation efficiency between the photogenerated electrons and holes in the *w*-TiO_2_ and *g*-TiO_2_ nanoparticles. The charge separation efficiency of photogenerated electrons and holes is a critical factor for the photoelectrode and photocatalytic activities^50–52^. Figure 6b shows EIS Nyquist plots of the *w*-TiO_2_ and *g*-TiO_2_ nanoparticles in the dark and under visible light irradiation. The arc radius of the EIS spectra reflects the interface layer resistance arising at the electrode surface^50^. A smaller arc radius indicates higher charge transfer efficiency^21,50–52^. The arc radius of the *g*-TiO_2_ nanoparticles was smaller than that of *w*-TiO_2_ nanoparticles in the dark and under visible light irradiation. This suggests that the *g*-TiO_2_ nanoparticles have a lower resistance than *w*-TiO_2_ nanoparticles, which can accelerate the interfacial charge-transfer processes. These observations are supported by the PL results. EIS further support the important role of Ti^3+^ and different types of defects and oxygen vacancies, which help improve the charge separation (electrons and holes) and transfer efficiency of photogenerated electrons and holes on the surface of the *g*-TiO_2_ nanoparticles compared to the *w*-TiO_2_ nanoparticles in the dark and under visible light irradiations.

### Visible light induced photocatalytic studies

**Figure 7:**
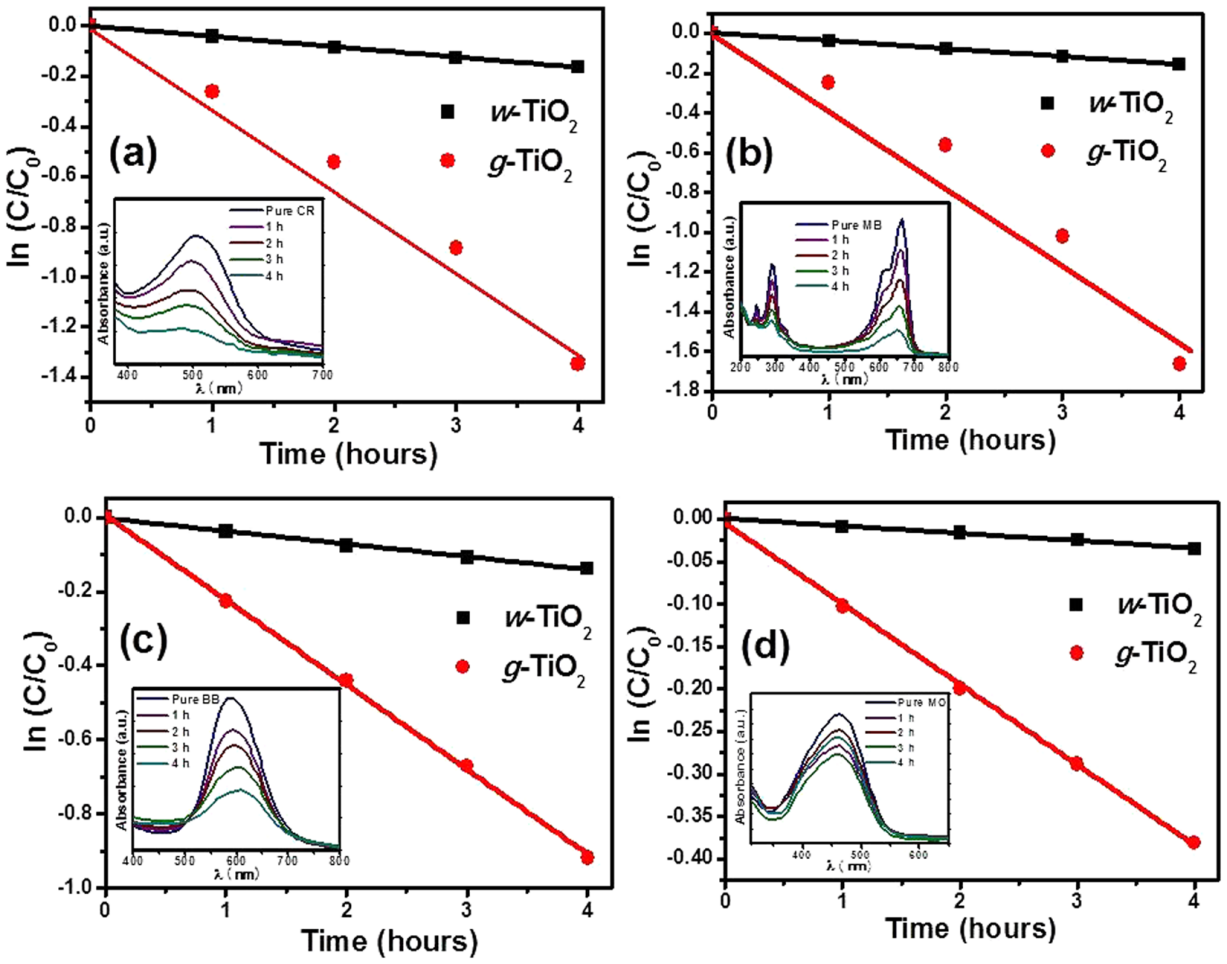
Visible light assisted photocatalytic degradation of (**a**) CR, (**b**) MB, (**c**) BB, and, (**d**) MO in the presence of the *w*-TiO_2_ and *g*-TiO_2_ nanoparticles.

The visible light-induced photocatalytic activities of the *w*-TiO_2_ and *g*-TiO_2_ nanoparticles were estimated by degrading CR, MB, BB and MO under the visible light (λ > 500 nm) as reported earlier^13,21,29,36,50^. The *g*-TiO_2_ nanoparticles showed better photocatalytic degradation of CR, MB, BB, and MO than *w*-TiO_2_ (Fig. 7). The visible light-induced photocatalytic degradation was estimated from the decrease in the absorption intensity of CR, MB, BB and MO at a fixed wavelength, λ_max_ = 492 nm, 665 nm, 588 nm, and 465 nm, respectively, during the course of the visible light-induced photocatalytic degradation experiment. The degradation was calculated using the relationship, ln C/C_0_ vs time (h) where C_0_ is the initial concentration and C is the concentration after visible light irradiation and degradation (Fig. 7). Inset of Fig. 7a,b,c, and d shows the respective decrease in absorbance after degradation. In addition, the degradation rate was also calculated to evaluate the precise degradation ability of the *w*-TiO_2_ and *g*-TiO_2_ nanoparticles. The rate constant (k) for the degradation of CR were 0.00072/h and 0.0570/h for *w*-TiO_2_ and *g*-TiO_2_ respectively, whereas 0.0018/h and 0.1203/h for degradation of MB by *w*-TiO_2_ and *g*-TiO_2_, respectively. Similarly, the k value for the degradation of BB was 0.0028/h and 0.02284/h for *w*-TiO_2_ and *g*-TiO_2_ respectively, whereas it was 0.0003/h and 0.0048/h for degradation of MO by *w*-TiO_2_ and *g*-TiO_2_, respectively. These results clearly show that *g*-TiO_2_ has higher k values for the degradation of CR, MB, BB, and MO compared to *w*-TiO_2_. The enhanced photocatalytic activity of the *g*-TiO_2_ nanoparticles compared to *w*-TiO_2_ can be explained by the surface modification and defects in *g*-TiO_2_ nanoparticles. Oxygen vacancies, other defects and Ti^3+^ centers enhance the photocatalytic activity^21,29^. The variation in the photocatalytic activity of *w*-TiO_2_ and *g*-TiO_2_ nanoparticles is also supported by DRS (Fig. 2(c)), EPR (Fig. 2b), and XPS (Figs 4 and 5). These outcomes evidently show that the visible light-induced photocatalytic performance of *g*-TiO_2_ nanoparticles can be amended greatly by reduction the band gap and making various defects and Ti^3+^ centers^4–9,21,29^ (Fig. 7).

**Figure 8:**
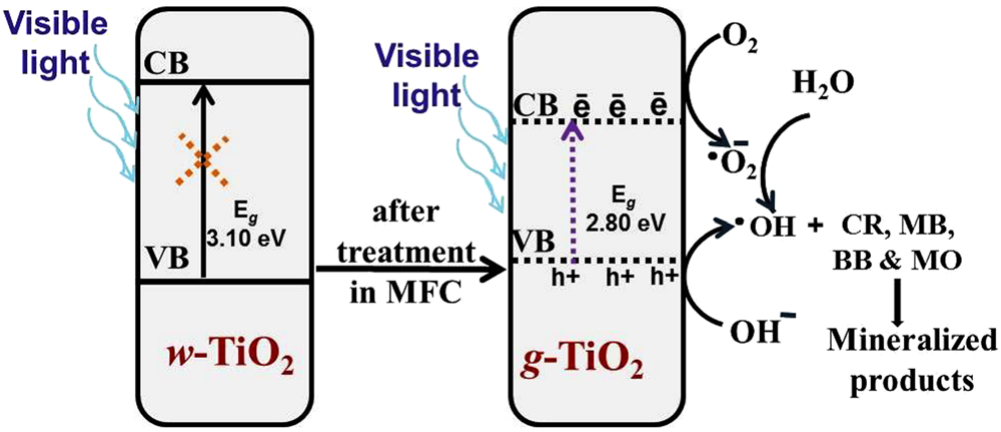
Proposed mechanism for the photocatalytic degradation of the dyes under visible light irradiation in the presence of *g*-TiO_2_ nanoparticles.

Figure 8 shows the proposed schematic mechanism for the Ti^3+^ and oxygen vacancy-induced visible light photocatalytic degradation of the colored dyes (CR, MB, BB, and MO) in the presence of *g*-TiO_2_ nanoparticles. The concept of heterogeneous photocatalysis is based on the ability of photocatalysts to harvest light energy that is required to generate electron–hole pairs for surface reactions. On the other hand, owing to the wide band gap of TiO_2_, it can only absorb UV light. Fortunately, the optical properties of TiO_2_ can be manipulated by defect engineering^4–9,13,21,29^. By introducing oxygen vacancies and other defects, the light absorption of TiO_2_ from UV can be extended to the visible region of the spectrum because the oxygen vacancies give rise to the local states below the conduction band edge. The as-formed oxygen vacancy states can participate in a new photoexcitation process. That is, the electron is excited to the oxygen vacancy states from the valence band with the energy of visible light, which gives rise to typical excitations in the visible region of the spectrum. For this reason, oxygen vacancies are called F centers^21^. In addition, the electrons remaining in the oxygen vacancies can also interact with the adjacent Ti^4+^ to give the Ti^3+^ species. The Ti^3+^ defects can also form a shallow donor level just below the conduction band, which can also contribute to the visible light response^8^. The enhancement in the performance of *g*-TiO_2_ was attributed to the high separation efficiency of e^–^/h^+^ pairs due to (Fig. 8) surface oxygen-vacancies and Ti^3+^ formation, which lead to band gap narrowing^4,5,21,26,29^. This band gap narrowing in *g*-TiO_2_ nanoparticles provides the visible light-induced photocatalytic activity. Band gap excitation of the semiconductor consequences in e^–^/h^+^ separation. The high oxidative potential of the holes in the photocatalyst permits the formation of reactive intermediates^21,29^. Reactive hydroxyl radicals (^•^OH) can be shaped either by the decay of water or by the reaction of a hole with OH^−^. The hydroxyl radicals and photogenerated holes are particularly strong, non-selective oxidants that prime to the degradation of CR, MB, BB, and MO at the surface of the *g*-TiO_2_ nanoparticles^4,5,21,29,30^.This can be accredited to the high concentration of oxygen vacancies, other defects, and Ti^3+^ centers formed in the *g*-TiO_2_ nanoparticles^21,29,38–45^ (Fig. 8).

In general, high temperature, pressure, and energy input are required during synthesis and modification of nanomaterials^4–9^. On the other hand, in this proposed method, the modification process was quite efficient because there was no any external energy input except mechanical stirring and modification process took place at room temperature under atmospheric pressure. During the TiO_2_ modification process in cathode of MFC, power density increased from 54.88 mW/m^2^ to 70.39 mW/m^2^ which is a big accomplishment^29^. There are few reports in which various types of materials have been used in cathode for the production of electricity with a high power density. For example, Han *et al*.^53^, reported simultaneous degradation of pollutants and power generation in a MFC, where gold nanoparticles acted as a catalyst. The authors reported that a 36.56 mW/m^2^ power density was achieved after complete removal of the pollutant. Therefore, in this study, the less expensive TiO_2_, which was modified for visible light photocatalytic applications without any energy input, was used as the cathode catalyst.

## Conclusions

A novel and biogenic method was used to modify the commercial TiO_2_ nanoparticles (*w*-TiO_2_) under ambient conditions, which resulted in an enhancement in their visible light assisted photocatalytic activities and photoelectrochemical behaviour. No structural changes occurred in *g*-TiO_2_, but Ti^3+^ formation, oxygen vacancies, and surface defects were identified. EIS and LSV in the dark and under visible light irradiation confirmed the visible light-induced photoactivity of the *g*-TiO_2_. Ti^3+^ and oxygen vacancy-induced visible light photocatalytic degradation of CR, MB, BB, and MO confirmed the improved photoactivity of the *g*-TiO_2_. This study suggests that *g*-TiO_2_ can be used as a visible light active photocatalyst as well as materials for photoelectrode. The MFC treatment can be employed to modify other metal oxides for enhanced visible light assisted photocatalytic activities and photoelectrochemical studies.

## Experimental procedures

### Materials used

TiO_2_ nanoparticles (size < 25 nm), methylene blue (MB), and brilliant blue (BB) were purchased from Sigma-Aldrich. Methyl orange (MO), Congo red (CR), and sodium sulphate were acquired from Duksan Pure Chemicals Co. Ltd. South Korea. Ethyl cellulose and α-terpineol were provided by KANTO Chemical Co., Japan and fluorine-doped transparent conducting oxide glass (FTO; F-doped SnO_2_ glass; 7 Ω/sq) was attained from Pilkington, USA. Altogether chemicals were of analytical grade and used as received. De-ionized water was prepared using a PURE ROUP 30 water purification system.

## Methods

UV-Vis diffuse reflectance/absorbance spectroscopy (DRS) of the powdered *w*-TiO_2_ and *g*-TiO_2_ nanoparticles was performed using an ultraviolet – visible – near infrared (UV-VIS-NIR) double beam spectrophotometer (VARIAN, Cary 5000, USA) equipped with a diffuse reflectance accessory. A He-Cd laser (Kimon, 1 K, Japan) with a wavelength of 325 nm and a power of 50 mW was used as the excitation source for the photoluminescence (PL) measurements. X-ray diffraction (XRD, PANalytical, X’pert PRO-MPD, The Netherlands) was performed using Cu Kα radiation (λ = 0.15405 nm). A Rietveld refinement was conducted using Muad 2.46 software. Raman spectroscopy was performed on a HR800 UV Raman microscope (Horiba Jobin-Yvon, France). The electron paramagnetic resonance (EPR) measurements were performed using a Bruker EMX system. X-ray photoelectron spectroscopy (XPS, ESCALAB 250 XPS System, Thermo Fisher Scientific U.K.) was conducted using the following X-ray source: monochromated Al Kα, h*ν* = 1486.6 eV, X-ray energy: 15 kV, 150 W and spot size: 500 μm. The XP spectra were fitted using the “Avantage program”. The microstructures of the *w*-TiO_2_ and *g*-TiO_2_ were observed by high resolution transmission electron microscopy (HRTEM, JEM-2100 JEOL) at an operating voltage of 200 kV combined with energy dispersive spectrometry (EDS) and a high angle annular dark field STEM (HAADF-STEM). The selected-area electron diffraction (SAED) images were recorded on a HRTEM instrument. The photocatalytic degradation and photoelectrochemical experiments (EIS and LSV) were conducted using a 400 W lamp with an irradiating intensity of 31.0 mWcm^−2^ (3 M, USA). The electrochemical impedance spectroscopy (EIS) and linear scan voltammetry (LSV) measurements were carried out using a potentiostat (Versa STAT 3, Princeton Research, USA) with a standard three-electrode system, in which Ag/AgCl (3.0 M KCl), a Pt gauge and fluorine-doped tin oxide (FTO) glass coated with *p*-TiO_2_, or *m*-TiO_2_ were used as the reference, counter, and working electrodes, respectively, in a 0.2 M Na_2_SO_4_ solution as the electrolyte. The working electrodes, with an effective area of 0.64 cm^2^, for EIS and LSV were prepared as follows. A 100 mg sample of each was suspended thoroughly by adding ethyl cellulose as a binder and α-terpineol as a solvent. The sample was mixed thoroughly for approximately 4 h and stirred at 50 °C for 4 h to obtain the paste. The obtained paste was coated on a FTO glass using the doctor-blade method, dried under a lamp for 24 h and used as the working electrode.

### Modification of TiO2 nanoparticles in Microbial Fuel Cell

Commercial TiO_2_ nanoparticles were modified in the cathode of the MFC. The MFC was setup as reported elsewhere^53^. A 200 mL aqueous dispersion of *w*-TiO_2_ (50 mM) was prepared in the cathode chamber of the MFC. The preliminary pH of the aqueous dispersions was 4.40. The MFC setup was run for 20 days. After modification, the final pH of the aqueous dispersion was 3.65. The almost white *w*-TiO_2_ changed to a light gray colour upon modification. The resultant dispersion was centrifuged and a greyish powder was obtained, dried in an oven at 80 °C for 24 h and used in the different characterization techniques and applications.

Before and during the modification process, the voltage generated by the MFC was monitored regularly and recorded using a computerized multimeter. After stabilizing the MFC, the voltage obtained was ~0.3514 V and after the modification of TiO_2_ in the MFC cathode, the voltage obtained was ~0.3979 V, which corresponds to a power density of 54.88 mW/m^2^ and 70.39 mW/m^2^, respectively.

### Photoelectrochemical studies of the w-TiO2 and g-TiO2 nanoparticles

To observe the photoelectrode performance of the *w*-TiO_2_ and *g*-TiO_2_ nanoparticles, photoelectrochemical experiments, such as EIS and LSV, were conducted under ambient conditions in the dark and under visible light irradiation in a 50 mL, 0.2 M Na_2_SO_4_ aqueous solution at room temperature. For each electrode, EIS was first performed in the dark and later under visible light irradiation (λ > 500 nm) at 0.0 V with frequencies ranging from 1–104 Hz. The photocurrent performance was attained by LSV in the dark and under visible light irradiation at a scan rate of 50 mV/s over the potential range, −1.0 to 1.0 V.

### Photocatalytic degradation of CR, MB, BB and MO using w-TiO2 and g-TiO2 nanoparticles

The photocatalytic performance of the *w*-TiO_2_ and *g*-TiO_2_ nanoparticles were confirmed for the photocatalytic degradation of CR (10 mg/L), MB (10 mg/L), BB (10 mg/L), and MO (10 mg/L), as reported elsewhere^21,29^. For the photodecomposition experiment, a 3.0 mg sample of each photocatalyst was suspended in 20 mL of the aqueous CR, MB, BB, and MO solutions. Every single solution was sonicated for 5 min and then stirred in the dark for 10 min to complete the adsorption and desorption equilibrium on the *w*-TiO_2_ and *g*-TiO_2_ nanoparticles. The solutions were irradiated with a 400 W lamp (λ > 500 nm) and the distance between the light source and dye solution was 25 cm. The four sets of experiments for CR, MB, BB, and MO degradation were observed for 4 h. The rates of CR, MB, BB, and MO degradation were examined by taking 1.7 mL of the samples from each set at every 1 h, centrifuging the degraded solution to remove the catalyst and recording the UV-vis spectra, from which the degradation of CR, MB, BB, and MO could be calculated.

As a control experiment, the *w*-TiO_2_ nanoparticles (reference photocatalyst, Sigma-Aldrich) were used to degrade CR, MB, BB, and MO under the similar experimental conditions. Every single degradation experiment was executed in triplicate to ensure the photocatalytic activity of the *w*-TiO_2_ and *g*-TiO_2_ nanoparticles. The stability and reusability of the *g*-TiO_2_ nanoparticles were also tested in a similar way to that stated above.

## Electronic supplementary material

Supplementary Info
