# Supplementary material for: Microbial fuel cell assisted band gap narrowed TiO2 for visible light-induced photocatalytic activities and power generation
Source: Sci Rep. 2018 Jan 29;8:1723. doi: 10.1038/s41598-018-19617-2 (PMC5788852; doi:10.1038/s41598-018-19617-2)
Supplement: Supplementary file 1 — Supplementary Info [file 41598_2018_19617_MOESM1_ESM.pdf]

Supplementary Information

Microbial fuel cell assisted band gap narrowed TiO_2 for visible light-induced activities and power generation

Mohammad Ehtisham Khan[1], Mohammad Mansoob Khan[2*], Bong-Ki Min[3], and Moo Hwan Cho[1*]

[1]*School of Chemical Engineering, Yeungnam University, Gyeongsan-si, Gyeongbuk 38541, South Korea.*
[2]*Chemical Sciences, Faculty of Science, Universiti Brunei Darussalam, Jalan Tungku Link, Gadong, BE 1410, Brunei Darussalam.*
[3]*Materials Science Centre, Yeungnam University, Gyeongsan-si, Gyeongbuk 38541, South Korea.*

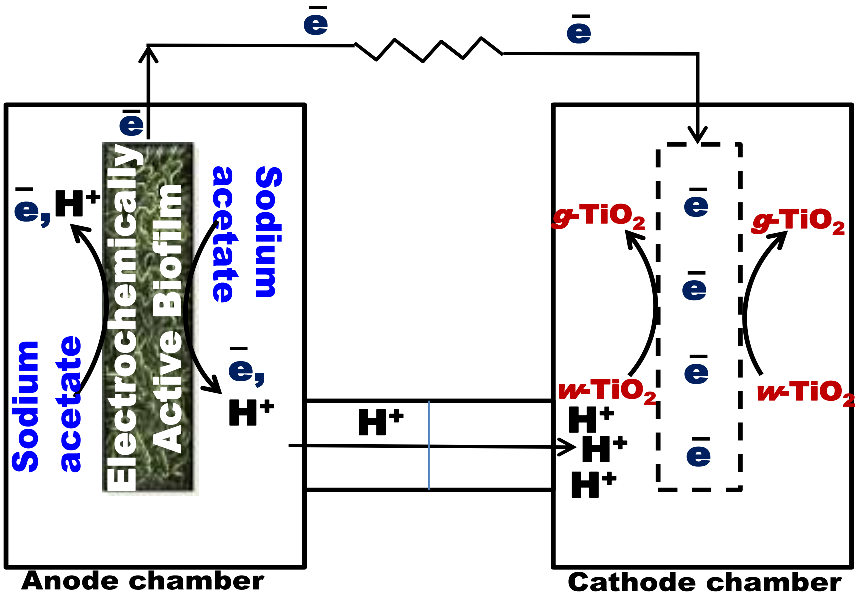

Fig. S1 Proposed schematic model for modification of the TiO_2 in MFC.

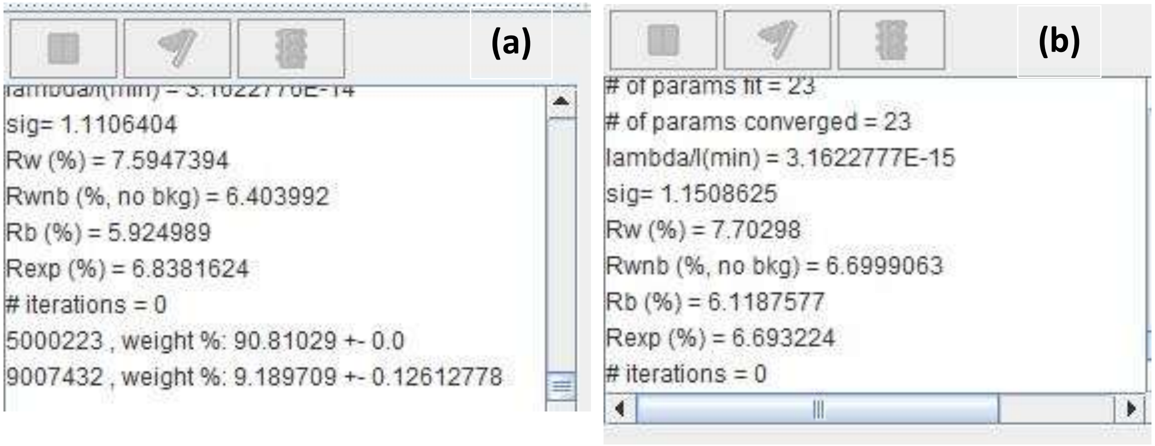

Fig. S2 Rietveld refinement indices for (**a**) *w*-TiO_2, and (**a**) *g*-TiO_2 nanoparticles.

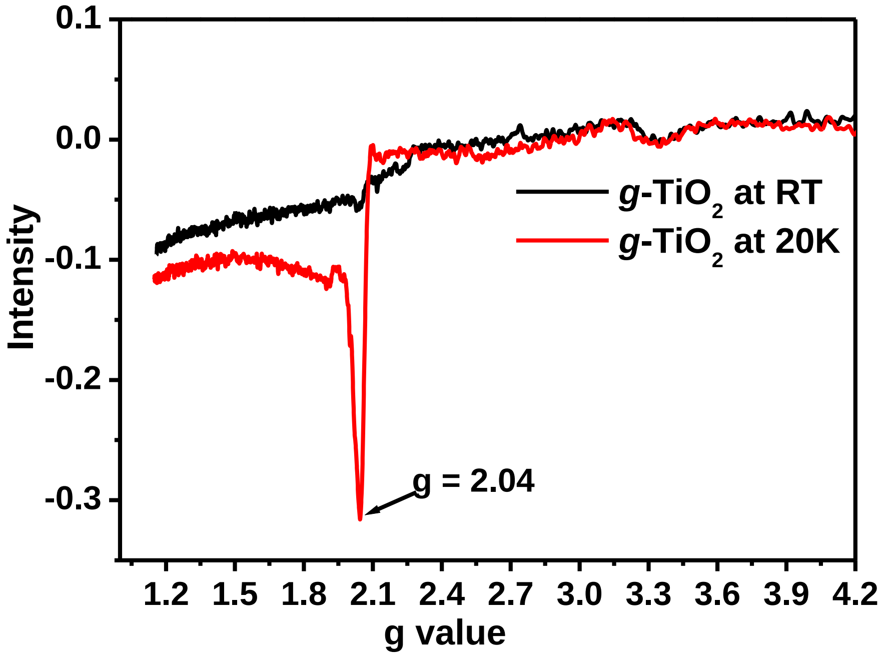

Fig. S3 g value of the *w*-TiO_2 and *g*-TiO_2 nanoparticles at RT and 20 K.

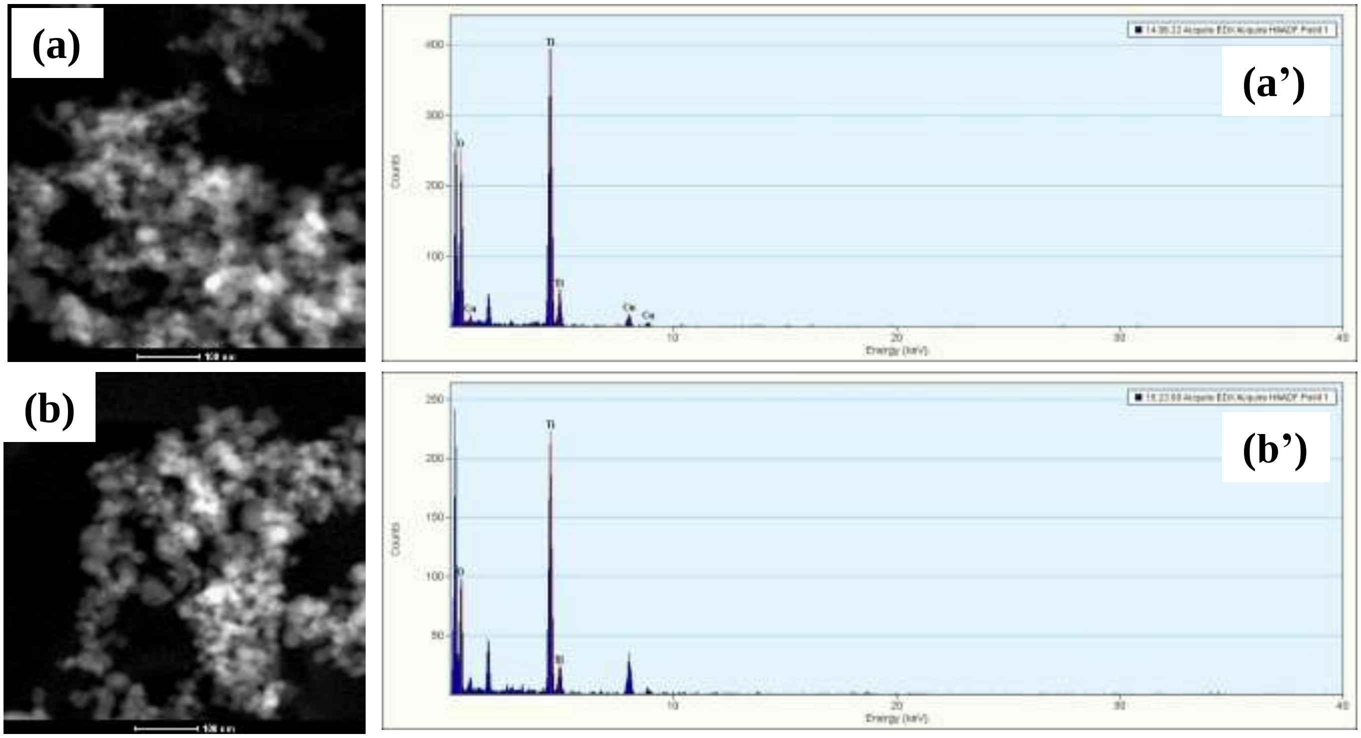

Fig. S4 Acquire HAADF (a and b) and EDX (a' and b') of *w*-TiO_2 and *g*-TiO_2 nanoparticles.

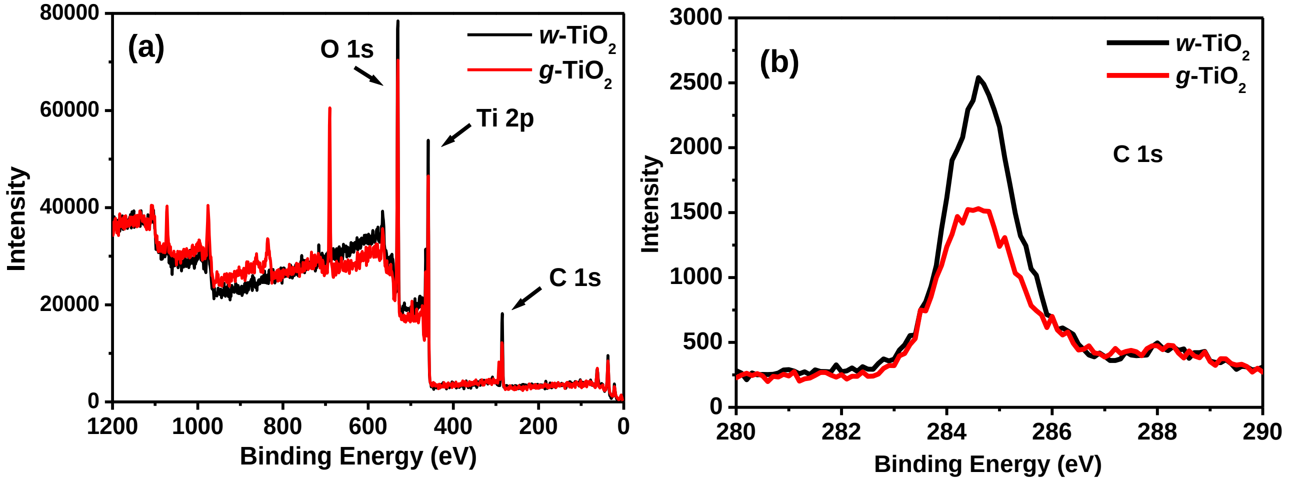

Fig. S5 XPS survey spectra of the *w*-TiO_2 and *g*-TiO_2 nanoparticles.
